# Genome Sequence of *Rhizobium ecuadorense* Strain CNPSo 671^T^, an Indigenous N_2_-Fixing Symbiont of the Ecuadorian Common Bean (*Phaseolus vulgaris* L.) Genetic Pool

**DOI:** 10.1128/genomeA.01058-15

**Published:** 2015-09-17

**Authors:** Renan Augusto Ribeiro, Jakeline Renata Marçon Delamuta, Douglas Fabiano Gomes, Renata Carolina Souza, Ligia Maria Oliveira Chueire, Mariangela Hungria

**Affiliations:** a CNPq, Brasília, Federal District, Brazil; b Embrapa Soja, Soil Biotechnology, Londrina, Paraná, Brazil; c CAPES, Brasília, Federal District, Brazil; d UFPR, Department of Microbiology, Curitiba, Paraná, Brazil

## Abstract

*Rhizobium ecuadorense* CNPSo 671^T^ was isolated from a common bean nodule in Ecuador. The draft genome brings novelty about indigenous rhizobial species in centers of genetic diversity of the legume.

## GENOME ANNOUNCEMENT

Common bean (*Phaseolus vulgaris* L.) is the most important legume cropped for human consumption worldwide, and the legume has the capacity of establishing N_2_-fixing symbioses with a broad range of rhizobial species (1). Genetic diversity of common bean rhizobia is high in South and Central Americas (1–5), but their N_2_-fixation effectiveness is highly affected by the plant, the rhizobium, and climatic and cropping conditions (1, 6, 7).

There are two major centers of genetic diversification of common bean, the Mesoamerican and the Andean (8), and in both *Rhizobium etli* has been found as the dominant microsymbiont (9, 10); however, some *R. etli* strains have been reclassified as *Rhizobium phaseoli* (11). The Peru-Ecuador genetic pool has also been proposed as another center of genetic diversification for common bean (12), and symbionts of the *R. phaseoli/R. etli*/*Rhizobium leguminosarum* clade have been isolated from this region (13). Genomes of rhizobia from the Peru-Ecuador genetic pool can help to enlighten our knowledge about the coevolution of the symbiosis.

Recently, a new species—*Rhizobium ecuadorense*—has been described for a lineage from Ecuador (14). Here, we report the draft genome of the type strain CNPSo 671^T^ (= UMR 1450^T^, PIMAMPIRS I 5^T^ = UMR 1450^T^ = LMG 27578^T^) of this new species, obtained from a common bean nodule in Pimampiro, Imbaburra, Ecuador.

To access the bacterial genome sequence, total DNA was extracted using the DNeasy blood and tissue kit (Qiagen) and processed on the MiSeq platform (Illumina) at Embrapa Soja, Londrina, Brazil. Ekhardt and genome analyses revealed that the strain has three plasmids and one chromosome.

Shotgun sequencing generated 4,964,249 paired-end reads (2 × 150 bp), corresponding to approximately 105-fold coverage. The FASTQ files were *de novo* assembled by Velvet (15). Genome annotation was performed in RAST (16), and the size was estimated at 7,386,509 bp with 1,922 contigs, 6,967 CDSs, and a G+C mol% content of 61.07. Annotation in RAST classified the sequences in 489 subsystems covering 45% of the genome. The major categories were of carbohydrates, amino acids, and derivatives; cofactors, vitamins, prosthetic groups, and pigments; protein metabolism; membrane transport; stress response; respiration; and fatty acids, lipids, and isoprenoids.

RAST identified the species *R. etli* bv. phaseoli CFN 42^T^ and *R. leguminosarum* bv. trifolii and bv. viciae as the closest neighbors of CNPSo 671^T^. Genes coding for Type I, II/IV, III, and IV secretion systems are present in the genome of CNPSo 671^T^, and several nodulation and nitrogen fixation genes showed 100% similarity with *R. phaseoli* CIAT 652. The results reinforce that *R. phaseoli* is an important symbiont of common bean and raise intriguing questions about coevolution with the host plant, as it has been proposed that the *R. phaseoli*–*R. etli* speciation process is more recent than that of other clades, such as *R. rhizogenes*–*R. tropici* (11).

### Nucleotide sequence accession number.

This whole-genome shotgun project has been deposited at DDBJ/EMBL/GenBank under the accession number LFIO00000000, SUBIC (SUB983818), Bioproject (PRJNA287284), BioSample (SAMN03779945). The version described in this paper is the first version.
